# Effects of the COVID-19 pandemic on mental health, working, and life situation of employees in the Swedish hospitality industry

**DOI:** 10.3389/fpubh.2023.1178847

**Published:** 2023-06-14

**Authors:** Kristin Feltmann, Nina-Katri J. Gustafsson, Tobias H. Elgán, Johanna Gripenberg, Pia Kvillemo

**Affiliations:** STAD (Stockholm Prevents Alcohol and Drug Problems), Centre for Psychiatry Research, Department of Clinical Neuroscience, Karolinska Institutet, Stockholm Health Care Services, Region Stockholm, Stockholm, Sweden

**Keywords:** nightlife, restaurants, worry, sadness, stress, COVID-19 crisis, income, staff

## Abstract

**Introduction:**

Previous studies reported that the coronavirus disease (COVID-19) pandemic has negatively affected the mental health of employees in the hospitality industry internationally, however, its effect in Sweden has not been studied. Unlike several other countries, Sweden never enforced a lockdown. Restaurants, bars, and hotels could remain open and host a limited number of guests but had to abide by certain restrictions.

**Methods:**

A cross-sectional survey was distributed among hospitality industry employees containing questions regarding the perceived effects of the pandemic on the respondents’ working and life situations and their physical and psychological health. The sample consisted of 699 individuals, with a response rate of 47.9%.

**Results:**

Although several respondents had been laid off or furloughed, the majority of the sample remained at the same employer. However, more than half of the respondents reported that their economic situation had deteriorated. Compared to before the pandemic, 38.1% experienced elevated levels of stress, 48.3% experienced elevated levels of worry, and 31.4% reported worsened mood. A deteriorating personal economy and difficulty in following COVID-19-related restrictions at work were associated with the worsening of these three mental health aspects. While the fear of becoming infected with COVID-19 was related to higher levels of stress, the fear of infecting others was related to higher levels of worry.

**Conclusion:**

Although Sweden imposed less strict measures than most other countries, the personal economy and mental health of hospitality workers were negatively affected by the COVID-19 pandemic.

## 1. Introduction

In March 2020, the World Health Organization (WHO) declared the COVID-19 outbreak a pandemic (1). A variety of measures were taken to prevent the spread of COVID-19; however, they differed between countries. Many countries, including several European countries, implemented travel restrictions and lockdowns of varying severity during several periods in 2020. Lockdowns ranged from forbidding individuals from exiting their residence unless they purchased groceries or medication, limiting the allowed human interactions, closing all shops except groceries and pharmacies, closing restaurants, nightclubs, schools, and universities, and requiring individuals to work or study from home (2, 3).

In contrast to other countries, few restrictions were implemented in Sweden, however, those that were implemented mainly affected the hospitality industry (Table 1). These restrictions were recommended by the Public Health Agency of Sweden and enforced by the Swedish government. Additionally, shops, restaurants, and bars in Sweden remained open but had to abide by restrictions that changed over time (Table 1). Examples of restrictions include allowing only table service, limiting the number of guests per table, defining the allowed distance between sitting groups, and limiting the time that alcohol could be served (4) (Table 1). In addition to these restrictions, the Public Health Agency issued several other recommendations, such as washing hands, maintaining distance, limiting social contacts, working from home if possible, staying at home when feeling unwell, older adults staying at home, and avoiding non-essential travel. Although Sweden changed the law to close preschools and schools in the case of special events, the Swedish government, in contrast to many other countries, never closed the schools during the COVID-19 pandemic. Sweden also introduced a regulation enabling online education, which many schools, especially secondary schools and universities, implemented (4).

**Table 1:** List and timeline of restrictions to prevent the spread of COVID-19 in Sweden.

Date	COVID-19 restrictions
2020	
12th March	A maximum of 500 people at public, cultural, and sports events
19th March	Travel restrictions outside the European Union (non-EU citizens)
25th March	Only table service at restaurants^1^
29th March	A maximum of 50 people at public events
30th March	Bans on visits to older adult care homes
18th April	New law enabling the closing down of restaurants^1^, shopping malls, etc., if needed (was never used and ended 1^st^ July)
1st July	New law regarding the responsibilities of restaurants^1^ to implement measures to reduce crowdedness and enable the Public Health Agency to directly impose restrictions on the establishments
7th July	A minimum of 1 m between sitting groups at restaurants^1^
1st November	A maximum of 300 people sitting down at gatherings (with 1 meter distance between them) A maximum of 50 people at dance events
8th November	A maximum of eight people per table at restaurants^1^
20th November	No alcohol service after 10 pm
24th November	A maximum of eight people at public events
21st December	Temporary travel restrictions from Denmark or the UK to Sweden
24th December	No alcohol service after 8 pm A maximum of four people per table at restaurants^1^
30th December	Demanding negative test results for COVID-19 for travelers from the UK
2021	
10th January	New law enabling the government to impose further restrictions
10th January	Limited number of guests for shops, gyms, and public pools (10 sqm/person)
1st March	Restaurants closing at 8:30 pm One person per table in restaurants within shopping malls (i.e., without own entrance)
3rd March	Demanding negative test results for COVID-19 from non-residents entering Sweden
1st June	A maximum of four people per table in restaurants^1^ within shopping malls Restaurants closing at 10:30 pm
1st July	Vaccination certificate or negative test results required for traveling within the EU A maximum of eight people per table at restaurants^1^ A maximum of 50 people at private events Lifted restrictions regarding limited opening hours for restaurants^1^
29th September	All restrictions lifted
23rd December	*Reintroduction of the following restrictions:* Only table service at restaurants^1^ with a maximum of eight people per table and distance between tables Vaccination certificates when the number of guests exceeds 500 10 sqm/person for indoor fairs, markets, and cultural events Demanding negative COVID-19 test for traveling into Sweden

^1^When referring to restaurants, other establishments serving food or alcohol (e.g., cafés and bars) were also included.

Restrictions on travel and gathering drastically reduced the number of guests in establishments in the hospitality industry and led to the closing of certain establishments, such as nightclubs. Due to the decline in demand, many individuals in the industry were laid off or furloughed. Estimations indicate that of the 200,000 individuals employed in the Swedish hospitality industry before the pandemic, over 50,000 lost their jobs and another 35,000 were laid off during the pandemic (5). During this period, no one knew how long the pandemic and its restrictions would last. This study hypothesized that this uncertainty, accompanied by the fear of becoming infected or infecting others with the coronavirus and the pandemic’s impact on their lives and working situations, may have negatively affected the mental health of employees in Sweden’s hospitality industry.

Qualitative studies explored the type of stress that the pandemic imposed on US hospitality industry employees, such as fear of infection, economic insecurity, isolation, and challenging work demands (6, 7). Several quantitative studies on hotel employees have indicated that these stressors have a negative effect on mental health and well-being (8–13). For example, a study conducted in Korea revealed that physical, mental, financial, and social concerns caused by the pandemic increased work stress, which, in turn, decreased well-being and mental health (8). Similarly, studies from Turkey, Pakistan, and the US revealed that the COVID-19 pandemic caused occupational stress that negatively affected the mental health and well-being of hotel employees and was related to higher absenteeism from work, decreased job satisfaction, and increased turnover (9–12).

In line with studies on hotel employees, it has also been shown that the pandemic negatively affected the mental health of restaurant employees (14–16). For example, a study in the US showed that employees who were furloughed had higher levels of psychological distress and substance and alcohol use than those who were still working or had been laid off (14). Another study in the US revealed that the fear of COVID-19 was associated with job insecurity and emotional exhaustion (15). However, a study among US restaurant employees showed that social and organizational support during the COVID-19 pandemic was associated with life satisfaction (16), indicating that increasing organizational support has the potential to increase employee well-being. In line with this, a recently published study among Romanian hotel and restaurant employees found that COVID-19-related occupational stress was positively associated with intentions to change jobs and that this association was hindered by organizational support (17). Nevertheless, the mental health of employees in the hospitality industry was negatively affected to a larger extent than that of employees in other industries during the pandemic, as shown in a recent study in the UK (18).

To prevent negative health effects in future crises, it is important, from a public health perspective, to increase knowledge about the effects of the COVID-19 pandemic on employees in the hospitality industry, since this industry employs many individuals. To the best of our knowledge, few studies have investigated the effects of the COVID-19 pandemic on the mental health of hospitality industry employees in Europe, and studies from Nordic countries are lacking. Sweden had no lockdown, and the restrictions mostly affected the hospitality industry. Therefore, this study aimed to investigate whether the COVID-19 pandemic has negatively affected the mental health, working, and life situations of hospitality industry employees.

## 2. Materials and methods

### 2.1. Procedure and participants

A cross-sectional survey was conducted after most restrictions had been lifted with the intent to collect data retrospectively. However, restrictions were reintroduced in the midterm of the data collection. This study intended to collect data by distributing paper surveys among participants in the Responsible Beverage Service (RBS) training, as this approach would reach many employees in the hospitality industry. However, during these restrictions, no physical courses were held; consequently, data collection was conducted online. Employers were contacted via mail, informed about the study, and asked to provide the e-mail addresses of employees. The online survey was distributed via e-mail links to employees working at hotels, bars, restaurants, and nightclubs in Stockholm. Informed consent was obtained from respondents before they responded to the survey. Four reminders were sent via e-mail between December 2021 and February 2022 before the survey was closed in March 2022. Between March and June 2022, data collection continued offline in a classroom setting using a paper-and-pen survey, including the same questions as those in the online survey. The participants were employees at licensed premises in Stockholm attending RBS training through the STAD (STockholm prevents Alcohol and Drug problems). RBS training is mandatory for staff working on licensed premises that open after 1 pm in Stockholm. The questionnaire was answered anonymously after a short introduction to the course, but before the main RBS content was presented. Participation was voluntary and the respondents provided informed consent by completing the questionnaire.

### 2.2. Measures

The survey comprised 30 questions covering five sections: (1) demographic information on sex, age, occupation, and work experience; (2) changes regarding work, economic, and living situations during the pandemic; (3) changes regarding mental health during the pandemic; (4) changes in alcohol use; and (5) COVID-19-related health questions, attitudes toward vaccinations, and COVID-19 guidelines.

### 2.3. Ethical approval

This study was reviewed and approved by the Ethical Review Board in Stockholm (Dnr 2021–06483-02).

### 2.4. Analysis

Participants working less than one year in the hospitality industry were excluded from the analysis (*n* = 12 online, *n* = 25 offline surveys), as the questions were retrospective. Descriptive statistics, presented as absolute and relative (%) numbers, illustrated the distribution of background characteristics; working, economic, and living situations; experiences of changes in mental health and somatic symptoms; alcohol drinking habits; illicit drug use; views on vaccination; fear of COVID-19 infection; experiences of COVID-19 symptoms; and difficulty following restrictions. Chi-square and t-tests were conducted to compare background, working, and economic characteristics and living situations between the online and offline samples.

To further investigate the potential factors predicting increased worry, stress, and low mood during the pandemic, four separate binomial logistic regression analyses were conducted. The dependent variable was dichotomized into worsened greatly/partly vs. no change, improved, or changed, independent of the pandemic. The four independent variables were dichotomized (0/1): worse economy, worse living situation, drinking more alcohol (worsened during the pandemic compared to before vs. no change/an improvement/a change unrelated to the pandemic), and difficulty adhering to restrictions (difficult/very difficult vs. very easy/easy/neither easy nor difficult). The significance level was set at *p* ≤ 0.05.

## 3. Results

### 3.1. Working and life situations among the online and offline samples

Of the 699 participants included in the sample, 335 responded, corresponding to a response rate of 47.9%. The response rates among those invited to participate in the online (*n* = 485) and offline (*n* = 214) surveys were 27.0 and 95.3%, respectively.

The online and offline samples were compared regarding background characteristics (Table 2) as well as changes in working, economic, and living situations during the pandemic (Table 3). Compared to the online sample, the offline sample consisted of slightly more males (*p* = 0.041), and the average age was 3 years younger (*p* = 0.038). Furthermore, a larger proportion of respondents in the offline sample worked in bars/pubs (*p* < 0.001) and restaurants (*p* < 0.001), and a smaller proportion worked in hotels (*p* < 0.001) than in the online sample. On average, the offline respondents worked three fewer years in the industry than the online respondents (*p* = 0.004).

**Table 2:** Descriptive background variables by sample.

	Total	Online (*n* = 119)	Offline (*n* = 179)	Statistics (Online/Offline)
	(*n* = 298)			
Sex, % (*n*)^a,b^				
Male	53.0 (157)	45.8 (54)	57.9 (103)	*χ*^2^ = 4.17, *p* = 0.041
Female	47.0 (139)	54.2 (64)	42.1 (75)	
Age (mean ± SD)^c^	31.3 ± 11.0	33.0 ± 12.2	30.2 ± 9.9	*t* = 2.09, *p* = 0.038
Nightlife industry, % (*n*)				
Bar/pub	45.3 (135)	23.5 (28)	59.8 (107)	*χ*^2^ = 37.90, p < 0.001
Nightclub	28.2 (84)	24.4 (29)	30.7 (55)	*χ*^2^ = 1.43, *p* = 0.232
Hotel	43.3 (129)	68.9 (82)	26.3 (47)	*χ*^2^ = 52.97, p < 0.001
Restaurant	55.4 (165)	39.5 (47)	65.9 (118)	*χ*^2^ = 20.20, p < 0.001
Student pub	2.0 (6)	0.8 (1)	2.8 (5)	*χ*^2^ = 1.38, *p* = 0.240
Other	4.4 (13)	0.8 (1)	6.7 (12)	*χ*^2^ = 5.89, *p* = 0.015
Years working in the nightlife industry	9.3 ± 8.6	11.1 ± 9.7	8.0 ± 7.5	*t* = 2.93, p = 0.004
(mean ± SD)^d^				

Data were missing for (a) *n* = 1 (online), (b) *n* = 1 (offline), (c) *n* = 4 (offline), and (d) *n* = 4 (offline).

**Table 3:** Working-, economic-, and living situation by sample (%).

	Total (*n* = 298) % (n)	Online (*n* = 119) % (n)	Offline (*n* = 179) % (n)	Statistics (Online/Offline)
Working situation^a,b^				
Same place, same role	38.9 (114)	38.1 (45)	39.4 (69)	*χ*^2^ = 0.05, *p* = 0.824
Same place, different role	13.7 (40)	15.3 (18)	12.6 (22)	*χ*^2^ = 0.43, *p* = 0.512
Furloughed	20.8 (61)	27.1 (32)	16.6 (29)	*χ*^2^ = 4.76, p = 0.029
Laid off	24.9 (73)	26.3 (31)	24.0 (42)	*χ*^2^ = 0.19, *p* = 0.659
New job within industry	29.0 (85)	28.8 (34)	29.1 (51)	*χ*^2^ = 0.00, *p* = 0.951
New job outside industry	8.5 (25)	9.3 (11)	8.0 (14)	*χ*^2^ = 0.16, *p* = 0.691
Started studying	8.9 (26)	5.9 (7)	10.9 (19)	*χ*^2^ = 2.11, *p* = 0.146
Economic situation^c,d^				
No change	32.4 (95)	38.5 (45)	28.4 (50)	*χ*^2^ (df = 4) = 8.93, *p* = 0.063
Worsened partly	36.5 (107)	40.2 (47)	34.1 (60)	
Worsened to a large extent	24.2 (71)	17.2 (20)	29.0 (51)	
Improved partly	5.1 (15)	3.4 (4)	6.3 (11)	
Improved to a large extent	1.7 (5)	0.9 (1)	2.3 (4)	
Living situation^e,f^				
No change	81.2 (238)	87.2 (102)	77.3 (136)	*χ*^2^ (df = 4) = 6.95, *p* = 0.139
Worsened	11.3 (33)	6.8 (8)	14.2 (25)	
Improved	1.0 (3)	1.7 (2)	0.6 (1)	
Changed, but neither improved nor worsened	3.4 (10)	1.7 (2)	4.5 (8)	
Changed, unrelated to pandemic	3.1 (9)	2.6 (3)	3.4 (6)	

(a) *n* = 1 (online), (b) *n* = 4 (offline), (c) *n* = 2 (online), (d) *n* = 3 (offline), (e) *n* = 2 (online), (f) *n* = 3 (offline).

The working situations were similar between the samples; however, more individuals were furloughed in the online sample than in the offline sample (*p* = 0.029). Most respondents continued to work in the same location during the pandemic. Nevertheless, 20.8% of the respondents had been furloughed and 24.9% were laid off during the pandemic. However, as the pandemic had been ongoing for 1.5–2 years at the time of data collection, several of these changes in working situations were valid for some participants. Changes in the economic situation did not significantly differ between the online and offline samples (*p* = 0.063). Overall, most participants reported that their economic situation had “worsened partly,” followed by “no change” and “worsened to a large extent.” Changes in living conditions did not significantly differ between the online and offline samples, with most respondents reporting no change in their living situations.

Overall, the online-offline comparisons described above deemed the samples to be fairly similar regarding background characteristics as well as changes in working, economic, and living situations; consequently, the remaining analyses did not separate online and offline samples.

### 3.2. Fear of infection and experienced COVID-19 symptoms

Of the participants, 12.3% were worried and 10.3% were very worried about becoming infected with the coronavirus. Moreover, 31.3% were worried and 21.6% were very worried about infecting others with the virus. Half of the participants tested positive for the virus and 71.3% had COVID-19 symptoms at some point during the pandemic. Approximately 15.2% of participants experienced long-term COVID-19 symptoms (>6 weeks). Of the respondents, 35.2% would not have received economic compensation if they had to stay home due to illness during the pandemic.

### 3.3. Effects on mental health and alcohol use

Approximately half of the participants did not experience changes in sleep or mood during the pandemic (Table 4). However, approximately one-third of the participants reported that their mood worsened during the pandemic. Furthermore, 38.1% reported higher levels of stress and 48.3% experienced higher levels of worry than before the pandemic.

**Table 4:** Mental health outcomes during the pandemic (%).

During the pandemic	Did not change % (n)	Worsened % (n)	Improved % (n)	Changed, but unrelated to pandemic % (n)
Sleep^a^	53.8 (157)	16.1 (47)	24.0 (70)	6.2 (18)
Stress^b^	41.8 (123)	38.1 (112)	14.6 (43)	5.1 (15)
Worry^c^	41.0 (121)	48.3 (142)	8.1 (24)	2.4 (7)
Mood^d^	51.9 (152)	31.4 (92)	11.3 (33)	5.5 (16)
Data was missing for (a) *n* = 6, (b) *n* = 5, (c) *n* = 4, and (d) *n* = 5.				

When participants reported increased worrying during the pandemic, they were asked about the potential reasons for their worry. Participants reported that their worry was related to their economic situation (67.2%), working situation (57.6%), other people’s health (42.4%), the pandemic in general (40.1%), their own health (38.4%), global health (20.3%), and the global economy (20.3%).

The majority (63.0%) of the participants (*n* = 292) reported that there was no change in their alcohol use compared to before the pandemic. However, 18.2% reported an increase in alcohol-drinking behavior, and 14.4% drank less alcohol during the pandemic.

### 3.4. Following COVID-19 restrictions in the hospitality industry

Most participants did not find it difficult to adhere to the restrictions in general (Table 5). It seemed that the participants found it most challenging to prevent crowding among guests, maintain the distance between guests, adhere to the mandated distance between tables, and limit the number of guests at each table and per establishment.

**Table 5:** Perceptions of how easy it was to follow the following guidelines (%).

How easy was it to follow the following guidelines	Very easy %	Easy %	Neither easy nor difficult %	Difficult %	Very Difficult %
Restrictions in general^a^	30.0	30.3	23.5	11.2	3.6
Stay home when you are sick^b^	39.4	27.0	13.1	13.5	5.7
Only table service^c^	24.5	26.0	18.7	17.2	8.1
Prevent crowdedness among guests^d^	15.3	23.6	18.9	25.1	14.5
Maintain distance between guests^e^	14.6	22.1	21.1	25.0	14.6
Mandated distance between tables^f^	23.6	29.3	22.5	14.3	6.1
Limit number of guests per establishment^g^	21.4	28.6	22.9	16.8	6.1
Limit number of guests per table^h^	20.9	28.4	21.6	16.5	9.0
Limited time of alcohol service^i^	28.0	28.7	22.6	7.9	8.6

Data were missing for (a) *n* = 21, (b) *n* = 16, (c) *n* = 25, (d) *n* = 27, (e) *n* = 18, (f) *n* = 18, (g) *n* = 18, (h) *n* = 19, and (i) *n* = 19.

The following numbers of people answered that the question was not relevant to their workplace: (a) *n* = 4, (b) *n* = 4, (c) *n* = 16, (d) *n* = 7, (e) *n* = 7, (f) *n* = 12, (g) *n* = 12, (h) *n* = 10, and (i) *n* = 12.

Of the respondents, 82.1% believed that the restrictions were communicated clearly within their organization, and 7.4 and 2.8% reported that they were communicated insufficiently or not at all, respectively. Furthermore, 17.0% believed that other restrictions should have been in place, such as wearing a mask, requiring a vaccination certificate, communicating that it was not the staff who decided on the restrictions, not allowing the premises to be open, and asking guests with symptoms to leave.

### 3.5. Factors associated with mental health problems

Among the respondents, 48.3% (*n* = 142) reported worsened levels of worry during the pandemic compared to before the pandemic. The results from the regression model, analyzing the predictive factors for increased worry, were overall statistically significant [X2 (13) = 32.1, *p* = 0.002, Nagelkerke R square = 0.154]. The analysis revealed that experiencing worsened personal economy significantly increased the probability of experiencing increased levels of worry. Furthermore, being worried about infecting others with COVID-19 and finding it difficult to adhere to restrictions increased the probability of being worried during the pandemic (Table 6). A similar regression analysis regarding worsened sleep was not significant [X2 (13) = 12.6, *p* = 0.483, Nagelkerke R square = 0.071]. The corresponding analyses for worsened stress [X2 (13) = 24.3, *p* = 0.028, Nagelkerke R square = 0.122] and mood [X2 (13) = 23.0, *p* = 0.041, Nagelkerke R square = 0.120] were significant. Worsened personal economy or difficulties adhering to restrictions increased the probability of experiencing increased levels of stress or a low mood. Furthermore, concerns about becoming infected with COVID-19 were associated with higher levels of stress.

**Table 6:** Binomial logistic regression of mental health outcomes and explanatory variables.

	Worry OR (95CI), value of *p*	Stress OR (95CI), value of *p*	Low mood OR (95CI), value of *p*
Years in the industry	1.02 (0.99–1.05), 0.307	0.99 (0.96–1.02), 0.577	1.01 (0.98–1.05), 0.503
Sex (male vs. female)	1.08 (0.64–1.83), 0.781	1.02 (0.60–1.75), 0.943	1.25 (0.71–2.21), 0.445
Worked in same role	0.94 (0.51–1.73), 0.837	1.11 (0.59–2.06), 0.754	1.19 (0.62–2.29), 0.607
Worked in diff. Role	0.78 (0.35–1.73), 0.540	0.65 (0.28–1.51), 0.320	1.02 (0.44–2.38), 0.963
Furloughed	0.77 (0.39–1.52), 0.451	1.15 (0.58–2.25), 0.693	0.83 (0.40–1.72), 0.621
Laid off	0.96 (0.47–1.96), 0.910	1.03 (0.51–2.09), 0.937	0.78 (0.37–1.66), 0.525
Worse economy	**2.80 (1.56–5.04), <0.001**	**2.18 (1.19–3.99), 0.012**	**2.61 (1.37–5.00), 0.004**
Worse living situation	0.99 (0.44–2.23), 0.984	0.51 (0.22–1.19), 0.119	1.13 (0.50–2.58), 0.764
Drank more alcohol	1.16 (0.60–2.28), 0.657	1.70 (0.87–3.31), 0.119	1.70 (0.86–3.39), 0.129
Worried to become infected with COVID-19	1.00 (0.50–2.01), 0.997	**2.45 (1.21–4.95), 0.012**	1.73 (0.84–3.57), 0.138
Worried to infect others with COVID-19	**2.16 (1.22–3.81), 0.008**	1.06 (0.59–1.89), 0.845	1.15 (0.62–2.13), 0.650
Difficulty following restrictions	**2.92 (1.33–6.41), 0.007**	**2.17 (1.03–4.59), 0.042**	**2.73 (1.28–5.81), 0.009**
Paid sick leave or compensation	1.19 (0.68–2.09), 0.550	0.83 (0.47–1.47), 0.529	0.84 (0.46–1.53), 0.575

The columns represent worsening mental health outcomes during the pandemic. Binomial regressions with DV (worsened during pandemic, improved/not changed/changed unrelated to the pandemic). The dependent variables were dichotomized into worsened greatly/partly vs. no change, improved, or changed, independent of the pandemic. The independent variables were dichotomized as follows: worse economy, worse living situation, and drinking more alcohol (worsened during the pandemic compared to before vs. no change/an improvement/a change unrelated to the pandemic) and difficulty following restrictions (difficult/very difficult vs. very easy/easy/neither easy nor difficult). Significant factors are highlighted in bold.

## 4. Discussion

In this study, employees in the hospitality industry (e.g., bars, restaurants, nightclubs, and hotels) responded to a survey on how 2 years of the COVID-19 pandemic affected their lives and mental health. A substantial number of respondents reported that the pandemic worsened their economic situation and that they experienced changes in their working situation, such as being furloughed or laid off. Approximately one-third of the respondents reported that their levels of stress or mood worsened during the pandemic, and nearly half of the participants experienced worsened levels of worry during the pandemic. The exploratory regression analysis revealed that worsened personal economy and difficulties following COVID-19 restrictions in the hospitality industry increased the probability of higher levels of worry, stress, and worsened mood during the pandemic. Furthermore, worry about infecting others with COVID-19 was related to higher levels of worry during the pandemic, and worry about becoming infected with COVID-19 was associated with higher levels of stress.

In contrast to other countries, Sweden did not impose lockdowns and restaurants and hotels remained open, however, they had to follow certain rules, which changed over time (4, 19). Consequently, the number of guests decreased drastically, resulting in decreased revenue and, therefore, layoffs (5). This study showed that several employees in the hospitality industry experienced worsened mental health due to the pandemic. Additionally, having experienced worsened personal economy increased the probability of also experiencing higher levels of worry, stress, and worsened mood during the pandemic compared to before the pandemic. While a previous study on restaurant employees, conducted in 2020, revealed that furloughed employees experienced worse levels of stress than those working or being laid off (14), the present study could not identify associations between job loss or furloughing and mental health outcomes. In addition, a study on Indian hospitality industry employees revealed that distress due to job loss, job freezing, and not being hired was associated with anxiety and depression (20). In general, job insecurity during the pandemic was related to work-related stress among hospitality industry employees in Malaysia (21) and the US (22). However, the current study was retrospective in nature over an extended period, during which, many individuals could have shifted between several states of employment. While the experience of being furloughed or laid off might negatively affect mental health, the present study indicated that personal economic situation, rather than employment status, affected levels of stress, worry, and mood. When asked what they worried about, 67.2 and 57.6% responded that they worried about their personal economy and work situation, respectively. In line with these findings, several qualitative studies on hospitality workers have demonstrated that financial concerns and economic insecurity negatively affect well-being (6–8). Furthermore, a study on hotel employees in Ghana indicated that the correlation between risk perception of COVID-19 and psychological distress was mediated by financial anxiety (23).

Loss of income can be very challenging for hospitality workers in Stockholm, as they are often young adults with little savings, often employed only part-time, and live in a city with relatively high living costs. Because the industry was doing well and constantly looking for staff before the pandemic, many employees were not members of the unemployment insurance fund. Statistics from immediately before the pandemic estimated that approximately 200,000 people were employed in the hospitality industry in Sweden; approximately 50,000 of them worked in Stockholm (24). In 2019, approximately 73,000 people in Sweden’s hospitality industry were members of the unemployment insurance fund, whereas this number rose to over 93,000 in 2020, and over 89,000 in 2021 (25). Hence, unless many employees were members of other unemployment insurance funds not specific to this industry during the pandemic, more than half of them would not have received economic support if they had lost their jobs. Economically, the pandemic harshly affected the hospitality industry and its employees, which consequently affected their mental well-being. Furthermore, as Baum et al. pointed out (26) one has to consider that employees of the hospitality industry generally have a lower educational, social and economic status compared to employees from other industries. These pre-existing differences could be connected to a vulnerability for mental health issues, which might have expressed themselves when the employment and economic situation worsened during the pandemic.

Difficulties in following restrictions were also related to worse mental health outcomes. In contrast to many other countries, which had several lockdowns closing many hospitality industry businesses, Sweden did not mandate the closure of most businesses. However, nightclubs were closed during most of the pandemic owing to restrictions such as only allowing table service, only allowing 50 people at dance events, and limiting the alcohol service time. The remaining businesses had to adapt to the restrictions at the time, which frequently changed (Table 1). The results indicated that respondents found it difficult to control guests’ behavior related to restrictions. In a qualitative study in the US, frontline workers, including those in the service industry, reported significant occupational stress due to customers not adhering to restrictions (27). In a study by Cuc et al., measures taken to prevent COVID-19 transmission and fear of becoming infected were related to occupational stress and increased turnover intentions (17). Turnover intentions among hospitality workers were associated with perceived job insecurity during the COVID-19 pandemic (28, 29), job satisfaction, workload, and pay, but not with coworker relationships (30). Similarly, commitment to the organization was negatively affected by COVID-19-induced stress among Korean hospitality workers (31). However, in another study on Korean hotel employees, COVID-19 event strength (a measure of the impact of the pandemic on organizations) was not correlated with turnover intention (32). Overall, these studies demonstrated that the pandemic caused significant stress in hospitality workers to the extent that they considered leaving their jobs. This is in line with the present findings, in which the pandemic and opposing measures and restrictions were related to feelings of worry, worsened mood, and stress. Moreover, while 12.3% were very worried and 10.3% were worried about becoming infected, only 17.0% wished for further measures such as wearing masks or vaccine certificates. In contrast to other countries, where these measures were obligatory in many environments accessible to the public from early on during the pandemic, the Swedish Public Health Agency *recommended* wearing masks during rush hours on public transport in January 2021 and *required* vaccine certificates for large events in December 2021 (4). An experimental study among hospitality workers in the United States found that employees felt grateful when communication regarding restrictions was in line with the Centers for Disease Control and Prevention’s guidelines. In contrast, they experienced anger against the organization if it was not (33). In the present study, opposing rules and restrictions were difficult for some individuals to manage, resulting in poor mental health. However, most respondents believed that the restrictions were clearly communicated within their organizations.

While the fear of becoming infected with COVID-19 was related to elevated stress levels, the fear of infecting others was related to higher levels of worry. Similarly, a study on hotel employees in Turkey found that COVID-19 stressors, such as fear of becoming infected, decreased mental well-being (9). Furthermore, a previous study in the United States found that the fear of contracting COVID-19 was positively correlated with job insecurity and emotional exhaustion among restaurant employees (15). Based on reports from the National Restaurant Association, the study found that two out of three restaurant employees in the United States lost their jobs during the pandemic. In contrast, the present study found that nearly half of the restaurant employees remained at the same employer, working either in the same or a different role. Moreover, although approximately one-third would not have received economic compensation when sick, the results for this variable were not significant in the regression analysis, including changes in worry or stress during the pandemic as explanatory variables.

### 4.1. Strengths and limitations

Participants were asked to recall and assess their working conditions and well-being over the past 2 years. Due to recall bias, self-reported data from an extended period are less reliable than reports from present situations, making the retrospective design a limitation of the current study. However, individuals may have forgotten problems more easily in cases where the mental health consequences experienced were less serious or only lasted for a short period. This study focused on estimation over a longer period. Although this approach may have ignored smaller problems, the problems reported in the study were either persistent or moderate to severe. Furthermore, the fear of becoming infected or infecting others could have potentially changed once people got vaccinated, which was not specifically investigated.

Additionally, a nearly equal distribution of males and females were recruited for the current study’s sample, with a mean age of 31 years, which is in line with reports from Swedish Statistics, a governmental agency, stating that employees in the hospitality industry are to equal extent men and women and are relatively young, with a mean age of 35 (24). Therefore, the current sample was representative of the study population. However, online and offline sampling methods revealed a larger response rate for the latter, as well as differences in demographics and the distribution of different types of establishments.

Another limitation of the study is that only employees in Stockholm were contacted. Although restrictions were implemented nationwide, the experiences in other cities or towns of various sizes might have differed from those in Stockholm. Nevertheless, it is possible that employees were affected more greatly in smaller places as the opportunity to change to another employer or other forms of employment or education is generally less in smaller cities or towns.

## 5. Conclusion

In contrast to several other countries, Sweden implemented no lockdowns during the COVID-19 pandemic, and restaurants and hotels remained open, albeit with restrictions. However, the restrictions affected the hospitality industry concerning the number of guests. This study found that hospitality industry employees experienced elevated levels of worry, stress, and low mood during the pandemic. Additionally, changes in the personal economic situation, as well as difficulties in conducting work following restrictions, could be associated with these mental health outcomes.

## Data availability statement

The raw data supporting the conclusions of this article will be made available by the authors, without undue reservation.

## Ethics statement

The studies involving human participants were reviewed and approved by the Ethical Review Board in Stockholm. The patients/participants provided their written informed consent to participate in this study.

## Author contributions

KF, TE, JG, and PK: study conception and design. KF, TE, and JG: data collection. KF, N-KG, and PK: analysis and interpretation of results. KF: writing original draft preparation. KF, N-KG, TE, JG, and PK: writing, review, and editing. All authors have read and approved the final version of the manuscript.

## Funding

This work was supported by the Public Health Agency of Sweden.

## Conflict of interest

The authors declare that the research was conducted in the absence of any commercial or financial relationships that could be construed as a potential conflict of interest.

## Publisher’s note

All claims expressed in this article are solely those of the authors and do not necessarily represent those of their affiliated organizations, or those of the publisher, the editors and the reviewers. Any product that may be evaluated in this article, or claim that may be made by its manufacturer, is not guaranteed or endorsed by the publisher.
